# Ten Simple Rules for Better Figures

**DOI:** 10.1371/journal.pcbi.1003833

**Published:** 2014-09-11

**Authors:** Nicolas P. Rougier, Michael Droettboom, Philip E. Bourne

**Affiliations:** 1 INRIA Bordeaux Sud-Ouest, Talence, France; 2 LaBRI, UMR 5800 CNRS, Talence, France; 3 Institute of Neurodegenerative Diseases, UMR 5293 CNRS, Bordeaux, France; 4 Space Telescope Science Institute, Baltimore, Maryland, United States of America; 5 Office of the Director, The National Institutes of Health, Bethesda, Maryland, United States of America

Scientific visualization is classically defined as the process of graphically displaying scientific data. However, this process is far from direct or automatic. There are so many different ways to represent the same data: scatter plots, linear plots, bar plots, and pie charts, to name just a few. Furthermore, the same data, using the same type of plot, may be perceived very differently depending on who is looking at the figure. A more accurate definition for scientific visualization would be a graphical interface between people and data. In this short article, we do not pretend to explain everything about this interface; rather, see [1], [2] for introductory work. Instead we aim to provide a basic set of rules to improve figure design and to explain some of the common pitfalls.

## Rule 1: Know Your Audience

**Figure 1:**
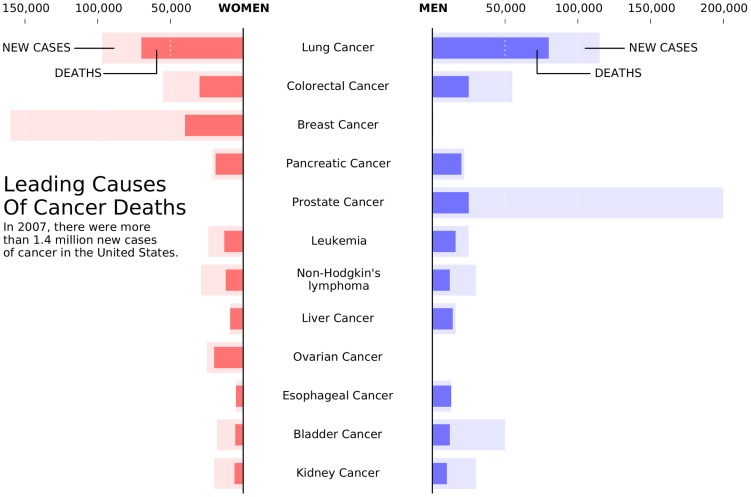
Know your audience. This is a remake of a figure that was originally published in the New York Times (NYT) in 2007. This new figure was made with matplotlib using approximated data. The data is made of four series (men deaths/cases, women deaths/cases) that could have been displayed using classical double column (deaths/cases) bar plots. However, the layout used here is better for the intended audience. It exploits the fact that the number of new cases is always greater than the corresponding number of deaths to mix the two values. It also takes advantage of the reading direction (English [left-to-right] for NYT) in order to ease comparison between men and women while the central labels give an immediate access to the main message of the figure (cancer). This is a self-contained figure that delivers a clear message on cancer deaths. However, it is not precise. The chosen layout makes it actually difficult to estimate the number of kidney cancer deaths because of its bottom position and the location of the labelled ticks at the top. While this is acceptable for a general-audience publication, it would not be acceptable in a scientific publication if actual numerical values were not given elsewhere in the article.

Given the definition above, problems arise when how a visual is perceived differs significantly from the intent of the conveyer. Consequently, it is important to identify, as early as possible in the design process, the audience and the message the visual is to convey. The graphical design of the visual should be informed by this intent. If you are making a figure for yourself and your direct collaborators, you can possibly skip a number of steps in the design process, because each of you knows what the figure is about. However, if you intend to publish a figure in a scientific journal, you should make sure your figure is correct and conveys all the relevant information to a broader audience. Student audiences require special care since the goal for that situation is to explain a concept. In that case, you may have to add extra information to make sure the concept is fully understood. Finally, the general public may be the most difficult audience of all since you need to design a simple, possibly approximated, figure that reveals only the most salient part of your research (Figure 1). This has proven to be a difficult exercise [3].

## Rule 2: Identify Your Message

**Figure 2:**
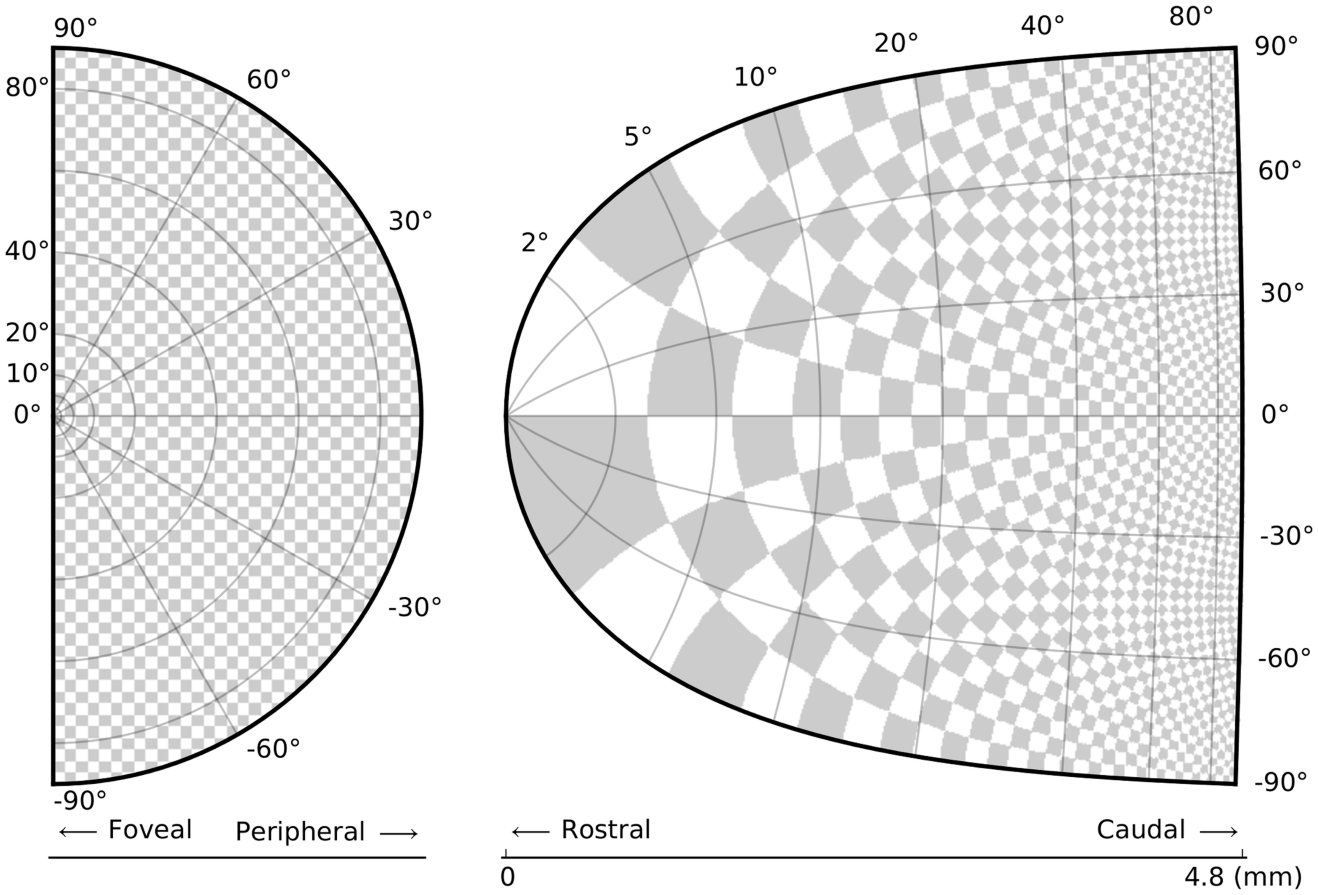
Identify your message. The superior colliculus (SC) is a brainstem structure at the crossroads of multiple functional pathways. Several neurophysiological studies suggest that the population of active neurons in the SC encodes the location of a visual target that induces saccadic eye movement. The projection from the retina surface (on the left) to the collicular surface (on the right) is based on a standard and quantitative model in which a logarithmic mapping function ensures the projection from retinal coordinates to collicular coordinates. This logarithmic mapping plays a major role in saccade decision. To better illustrate this role, an artificial checkerboard pattern has been used, even though such a pattern is not used during experiments. This checkerboard pattern clearly demonstrates the extreme magnification of the foveal region, which is the main message of the figure.

A figure is meant to express an idea or introduce some facts or a result that would be too long (or nearly impossible) to explain only with words, be it for an article or during a time-limited oral presentation. In this context, it is important to clearly identify the role of the figure, i.e., what is the underlying message and how can a figure best express this message? Once clearly identified, this message will be a strong guide for the design of the figure, as shown in Figure 2. Only after identifying the message will it be worth the time to develop your figure, just as you would take the time to craft your words and sentences when writing an article only after deciding on the main points of the text. If your figure is able to convey a striking message at first glance, chances are increased that your article will draw more attention from the community.

## Rule 3: Adapt the Figure to the Support Medium

**Figure 3:**
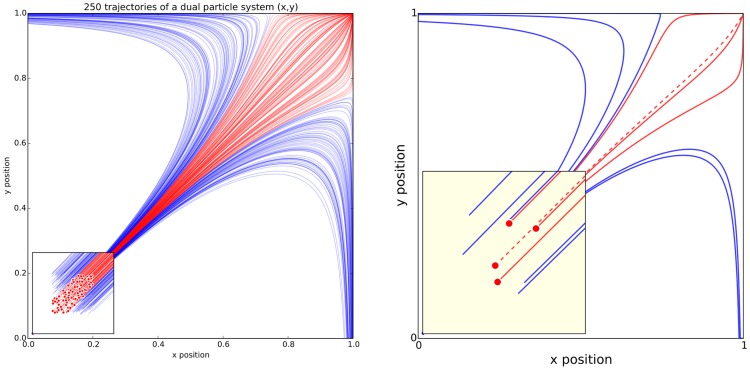
Adapt the figure to the support medium. These two figures represent the same simulation of the trajectories of a dual-particle system (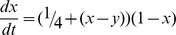, 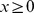, 
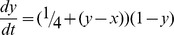
, 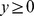) where each particle interacts with the other. Depending on the initial conditions, the system may end up in three different states. The left figure has been prepared for a journal article where the reader is free to look at every detail. The red color has been used consistently to indicate both initial conditions (red dots in the zoomed panel) and trajectories (red lines). Line transparency has been increased in order to highlight regions where trajectories overlap (high color density). The right figure has been prepared for an oral presentation. Many details have been removed (reduced number of trajectories, no overlapping trajectories, reduced number of ticks, bigger axis and tick labels, no title, thicker lines) because the time-limited display of this figure would not allow for the audience to scrutinize every detail. Furthermore, since the figure will be described during the oral presentation, some parts have been modified to make them easier to reference (e.g., the yellow box, the red dashed line).

A figure can be displayed on a variety of media, such as a poster, a computer monitor, a projection screen (as in an oral presentation), or a simple sheet of paper (as in a printed article). Each of these media represents different physical sizes for the figure, but more importantly, each of them also implies different ways of viewing and interacting with the figure. For example, during an oral presentation, a figure will be displayed for a limited time. Thus, the viewer must quickly understand what is displayed and what it represents while still listening to your explanation. In such a situation, the figure must be kept simple and the message must be visually salient in order to grab attention, as shown in Figure 3. It is also important to keep in mind that during oral presentations, figures will be video-projected and will be seen from a distance, and figure elements must consequently be made thicker (lines) or bigger (points, text), colors should have strong contrast, and vertical text should be avoided, etc. For a journal article, the situation is totally different, because the reader is able to view the figure as long as necessary. This means a lot of details can be added, along with complementary explanations in the caption. If we take into account the fact that more and more people now read articles on computer screens, they also have the possibility to zoom and drag the figure. Ideally, each type of support medium requires a different figure, and you should abandon the practice of extracting a figure from your article to be put, as is, in your oral presentation.

## Rule 4: Captions Are Not Optional

Whether describing an experimental setup, introducing a new model, or presenting new results, you cannot explain everything within the figure itself—a figure should be accompanied by a caption. The caption explains how to read the figure and provides additional precision for what cannot be graphically represented. This can be thought of as the explanation you would give during an oral presentation, or in front of a poster, but with the difference that you must think in advance about the questions people would ask. For example, if you have a bar plot, do not expect the reader to guess the value of the different bars by just looking and measuring relative heights on the figure. If the numeric values are important, they must be provided elsewhere in your article or be written very clearly on the figure. Similarly, if there is a point of interest in the figure (critical domain, specific point, etc.), make sure it is visually distinct but do not hesitate to point it out again in the caption.

## Rule 5: Do Not Trust the Defaults

**Figure 4:**
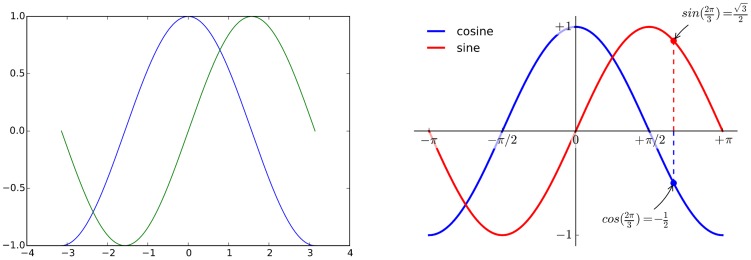
Do not trust the defaults. The left panel shows the sine and cosine functions as rendered by matplotlib using default settings. While this figure is clear enough, it can be visually improved by tweaking the various available settings, as shown on the right panel.

Any plotting library or software comes with a set of default settings. When the end-user does not specify anything, these default settings are used to specify size, font, colors, styles, ticks, markers, etc. (Figure 4). Virtually any setting can be specified, and you can usually recognize the specific style of each software package (Matlab, Excel, Keynote, etc.) or library (LaTeX, matplotlib, gnuplot, etc.) thanks to the choice of these default settings. Since these settings are to be used for virtually any type of plot, they are not fine-tuned for a specific type of plot. In other words, they are good enough for any plot but they are best for none. All plots require at least some manual tuning of the different settings to better express the message, be it for making a precise plot more salient to a broad audience, or to choose the best colormap for the nature of the data. For example, see [4] for how to go from the default settings to a nicer visual in the case of the matplotlib library.

## Rule 6: Use Color Effectively

**Figure 5:**
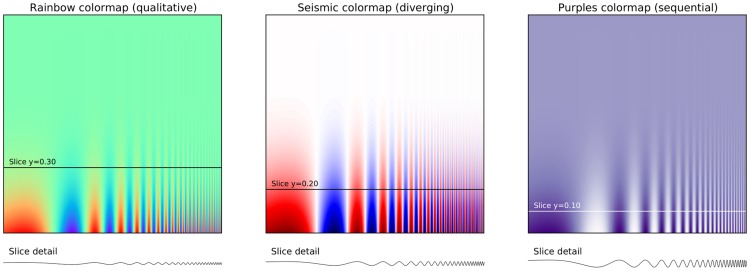
Use color effectively. This figure represents the same signal, whose frequency increases to the right and intensity increases towards the bottom, using three different colormaps. The rainbow colormap (qualitative) and the seismic colormap (diverging) are equally bad for such a signal because they tend to hide details in the high frequency domain (bottom-right part). Using a sequential colormap such as the purple one, it is easier to see details in the high frequency domain. Adapted from [5].

Color is an important dimension in human vision and is consequently equally important in the design of a scientific figure. However, as explained by Edward Tufte [1], color can be either your greatest ally or your worst enemy if not used properly. If you decide to use color, you should consider which colors to use and where to use them. For example, to highlight some element of a figure, you can use color for this element while keeping other elements gray or black. This provides an enhancing effect. However, if you have no such need, you need to ask yourself, “Is there any reason this plot is blue and not black?” If you don't know the answer, just keep it black. The same holds true for colormaps. Do not use the default colormap (e.g., jet or rainbow) unless there is an explicit reason to do so (see Figure 5 and [5]). Colormaps are traditionally classified into three main categories:

- *Sequential*: one variation of a unique color, used for quantitative data varying from low to high.
- *Diverging*: variation from one color to another, used to highlight deviation from a median value.
- *Qualitative*: rapid variation of colors, used mainly for discrete or categorical data.

Use the colormap that is the most relevant to your data. Lastly, avoid using too many similar colors since color blindness may make it difficult to discern some color differences (see [6] for detailed explanation).

## Rule 7: Do Not Mislead the Reader

**Figure 6:**
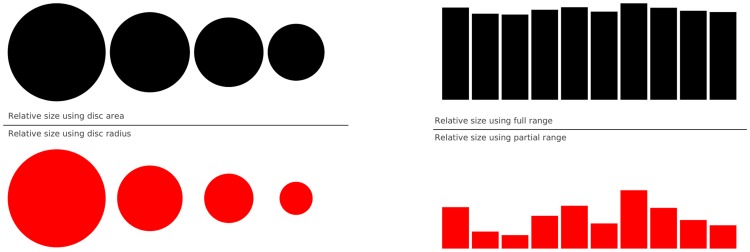
Do not mislead the reader. On the left part of the figure, we represented a series of four values: 30, 20, 15, 10. On the upper left part, we used the disc area to represent the value, while in the bottom part we used the disc radius. Results are visually very different. In the latter case (red circles), the last value (10) appears very small compared to the first one (30), while the ratio between the two values is only 3∶1. This situation is actually very frequent in the literature because the command (or interface) used to produce circles or scatter plots (with varying point sizes) offers to use the radius as default to specify the disc size. It thus appears logical to use the value for the radius, but this is misleading. On the right part of the figure, we display a series of ten values using the full range for values on the top part (y axis goes from 0 to 100) or a partial range in the bottom part (y axis goes from 80 to 100), and we explicitly did not label the y-axis to enhance the confusion. The visual perception of the two series is totally different. In the top part (black series), we tend to interpret the values as very similar, while in the bottom part, we tend to believe there are significant differences. Even if we had used labels to indicate the actual range, the effect would persist because the bars are the most salient information on these figures.

What distinguishes a scientific figure from other graphical artwork is the presence of data that needs to be shown as objectively as possible. A scientific figure is, by definition, tied to the data (be it an experimental setup, a model, or some results) and if you loosen this tie, you may unintentionally project a different message than intended. However, representing results objectively is not always straightforward. For example, a number of implicit choices made by the library or software you're using that are meant to be accurate in most situations may also mislead the viewer under certain circumstances. If your software automatically re-scales values, you might obtain an objective representation of the data (because title, labels, and ticks indicate clearly what is actually displayed) that is nonetheless visually misleading (see bar plot in Figure 6); you have inadvertently misled your readers into visually believing something that does not exist in your data. You can also make explicit choices that are wrong by design, such as using pie charts or 3-D charts to compare quantities. These two kinds of plots are known to induce an incorrect perception of quantities and it requires some expertise to use them properly. As a rule of thumb, make sure to always use the simplest type of plots that can convey your message and make sure to use labels, ticks, title, and the full range of values when relevant. Lastly, do not hesitate to ask colleagues about their interpretation of your figures.

## Rule 8: Avoid “Chartjunk”

**Figure 7:**
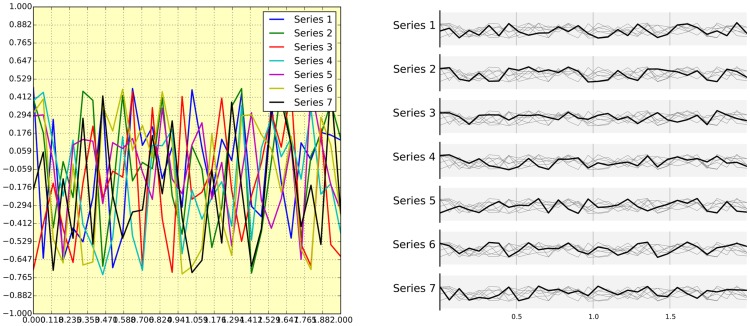
Avoid chartjunk. We have seven series of samples that are equally important, and we would like to show them all in order to visually compare them (exact signal values are supposed to be given elsewhere). The left figure demonstrates what is certainly one of the worst possible designs. All the curves cover each other and the different colors (that have been badly and automatically chosen by the software) do not help to distinguish them. The legend box overlaps part of the graphic, making it impossible to check if there is any interesting information in this area. There are far too many ticks: x labels overlap each other, making them unreadable, and the three-digit precision does not seem to carry any significant information. Finally, the grid does not help because (among other criticisms) it is not aligned with the signal, which can be considered discrete given the small number of sample points. The right figure adopts a radically different layout while using the same area on the sheet of paper. Series have been split into seven plots, each of them showing one series, while other series are drawn very lightly behind the main one. Series labels have been put on the left of each plot, avoiding the use of colors and a legend box. The number of x ticks has been reduced to three, and a thin line indicates these three values for all plots. Finally, y ticks have been completely removed and the height of the gray background boxes indicate the [−1,+1] range (this should also be indicated in the figure caption if it were to be used in an article).

Chartjunk refers to all the unnecessary or confusing visual elements found in a figure that do not improve the message (in the best case) or add confusion (in the worst case). For example, chartjunk may include the use of too many colors, too many labels, gratuitously colored backgrounds, useless grid lines, etc. (see left part of Figure 7). The term was first coined by Edward Tutfe in [1], in which he argues that any decorations that do not tell the viewer something new must be banned: “Regardless of the cause, it is all non-data-ink or redundant data-ink, and it is often chartjunk.” Thus, in order to avoid chartjunk, try to save ink, or electrons in the computing era. Stephen Few reminds us in [7] that graphs should ideally “represent all the data that is needed to see and understand what's meaningful.” However, an element that could be considered chartjunk in one figure can be justified in another. For example, the use of a background color in a regular plot is generally a bad idea because it does not bring useful information. However, in the right part of Figure 7, we use a gray background box to indicate the range [−1,+1] as described in the caption. If you're in doubt, do not hesitate to consult the excellent blog of Kaiser Fung [8], which explains quite clearly the concept of chartjunk through the study of many examples.

## Rule 9: Message Trumps Beauty

**Figure 8:**
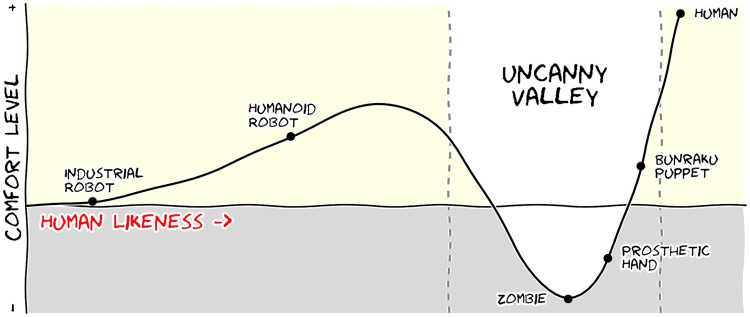
Message trumps beauty. This figure is an extreme case where the message is particularly clear even if the aesthetic of the figure is questionable. The uncanny valley is a well-known hypothesis in the field of robotics that correlates our comfort level with the human-likeness of a robot. To express this hypothetical nature, hypothetical data were used (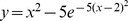) and the figure was given a sketched look (xkcd filter on matplotlib) associated with a cartoonish font that enhances the overall effect. Tick labels were also removed since only the overall shape of the curve matters. Using a sketch style conveys to the viewer that the data is approximate, and that it is the higher-level concepts rather than low-level details that are important [10].

Figures have been used in scientific literature since antiquity. Over the years, a lot of progress has been made, and each scientific domain has developed its own set of best practices. It is important to know these standards, because they facilitate a more direct comparison between models, studies, or experiments. More importantly, they can help you to spot obvious errors in your results. However, most of the time, you may need to design a brand-new figure, because there is no standard way of describing your research. In such a case, browsing the scientific literature is a good starting point. If some article displays a stunning figure to introduce results similar to yours, you might want to try to adapt the figure for your own needs (note that we did not say copy; be careful with image copyright). If you turn to the web, you have to be very careful, because the frontiers between data visualization, infographics, design, and art are becoming thinner and thinner [9]. There exists a myriad of online graphics in which aesthetic is the first criterion and content comes in second place. Even if a lot of those graphics might be considered beautiful, most of them do not fit the scientific framework. Remember, in science, message and readability of the figure is the most important aspect while beauty is only an option, as dramatically shown in Figure 8.

## Rule 10: Get the Right Tool

There exist many tools that can make your life easier when creating figures, and knowing a few of them can save you a lot of time. Depending on the type of visual you're trying to create, there is generally a dedicated tool that will do what you're trying to achieve. It is important to understand at this point that the software or library you're using to make a visualization can be different from the software or library you're using to conduct your research and/or analyze your data. You can always export data in order to use it in another tool. Whether drawing a graph, designing a schema of your experiment, or plotting some data, there are open-source tools for you. They're just waiting to be found and used. Below is a small list of open-source tools.

**Matplotlib** is a python plotting library, primarily for 2-D plotting, but with some 3-D support, which produces publication-quality figures in a variety of hardcopy formats and interactive environments across platforms. It comes with a huge gallery of examples that cover virtually all scientific domains (http://matplotlib.org/gallery.html).

**R** is a language and environment for statistical computing and graphics. R provides a wide variety of statistical (linear and nonlinear modeling, classical statistical tests, time-series analysis, classification, clustering, etc.) and graphical techniques, and is highly extensible.

**Inkscape** is a professional vector graphics editor. It allows you to design complex figures and can be used, for example, to improve a script-generated figure or to read a PDF file in order to extract figures and transform them any way you like.

**TikZ and PGF** are TeX packages for creating graphics programmatically. TikZ is built on top of PGF and allows you to create sophisticated graphics in a rather intuitive and easy manner, as shown by the Tikz gallery (http://www.texample.net/tikz/examples/all/).

**GIMP** is the GNU Image Manipulation Program. It is an application for such tasks as photo retouching, image composition, and image authoring. If you need to quickly retouch an image or add some legends or labels, GIMP is the perfect tool.

**ImageMagick** is a software suite to create, edit, compose, or convert bitmap images from the command line. It can be used to quickly convert an image into another format, and the huge script gallery (http://www.fmwconcepts.com/imagemagick/index.php) by Fred Weinhaus will provide virtually any effect you might want to achieve.

**D3.js** (or just D3 for Data-Driven Documents) is a JavaScript library that offers an easy way to create and control interactive data-based graphical forms which run in web browsers, as shown in the gallery at http://github.com/mbostock/d3/wiki/Gallery.

**Cytoscape** is a software platform for visualizing complex networks and integrating these with any type of attribute data. If your data or results are very complex, cytoscape may help you alleviate this complexity.

**Circos** was originally designed for visualizing genomic data but can create figures from data in any field. Circos is useful if you have data that describes relationships or multilayered annotations of one or more scales.

## Notes

All the figures for this article were produced using matplotlib, and figure scripts are available from https://github.com/rougier/ten-rules.

## References

[1] Tufte EG (1983) The Visual Display of Quantitative Information. Cheshire, Connecticut: Graphics Press.

[2] Doumont JL (2009) Trees, maps, and theorems. Brussels: Principiae.

[3] Kosara R, Mackinlay J (2013) Storytelling: The next step for visualization. IEEE Comput 46: 44–50.

[4] Rougier NP (2012) Scientific visualization and matplotlib tutorial. Euroscipy 2012 & 2013. Available: http://www.loria.fr/~rougier/teaching/matplotlib/matplotlib.html. Accessed 12 August 2014.

[5] Borland D, Taylor RM (2007) Rainbow color map (still) considered harmful. IEEE Comput Graph Appl 27: 14–17. 17388198 10.1109/mcg.2007.323435

[6] Okabe M, Ito K (2008). Color universal design (cud) - how to make figures and presentations that are friendly to colorblind people. Available: http://jfly.iam.u-tokyo.ac.jp/color/. Accessed 12 August 2014.

[7] Few S (2011) The chartjunk debate, a close examination of recent findings. Visual Business Intelligence Newsletter. Available: http://www.perceptualedge.com/articles/visual_business_intelligence/the_chartjunk_debate.pdf. Accessed 12 August 2014.

[8] Fung K (2005). Junk charts: Recycling chartjunk as junk art. Available: http://junkcharts.typepad.com. Accessed 12 August 2014.

[9] Borkin MA, Vo AA, Bylinskii Z, Isola P, Sunkavalli S, et al (2013) What makes a visualization memorable? IEEE Trans Vis Comput Graph 19: 2306–2315. 24051797 10.1109/TVCG.2013.234

[10] Schumann J, Strothotte T, Raab A, Laser S (1996) Assessing the effect of non-photorealistic rendered images in cad. In: Proceedings of the SIGCHI Conference on Human Factors in Computing Systems; 13–18 April 1996; New York, New York, United States. CHI 96. New York: Association for Computing Machinery. pp.35–41.

